# Game-Based eHealth Interventions for the Reduction of Fatigue in People With Chronic Diseases: Systematic Review and Meta-Analysis

**DOI:** 10.2196/55034

**Published:** 2024-10-17

**Authors:** Leonie S Warlo, Souraya El Bardai, Andrica de Vries, Marie-Lise van Veelen, Suzan Moors, Edmond HHM Rings, Jeroen S Legerstee, Bram Dierckx

**Affiliations:** 1 Department of Child and Adolescent Psychiatry/Psychology Sophia Children’s Hospital Erasmus Medical Center Rotterdam Netherlands; 2 Department of Pediatric Oncology Sophia Children’s Hospital Erasmus Medical Center Rotterdam Netherlands; 3 Princess Máxima Center for Pediatric Oncology Utrecht Netherlands; 4 Department of Neurosurgery Sophia Children's Hospital Erasmus Medical Center Rotterdam Netherlands; 5 Department of Physiotherapy Sophia Children's Hospital Erasmus Medical Center Rotterdam Netherlands; 6 Department of Pediatrics Sophia Children’s Hospital Erasmus Medical Center Rotterdam Netherlands; 7 Levvel Academic Center for Child and Adolescent Psychiatry and Specialized Youth Care Amsterdam Netherlands; 8 Research Institute of Child Development and Education University of Amsterdam Amsterdam Netherlands

**Keywords:** fatigue, chronic disease, eHealth, serious games, exergames

## Abstract

**Background:**

Fatigue is a common and debilitating side effect of chronic diseases, significantly impacting patients’ quality of life. While physical exercise and psychological treatments have been shown to reduce fatigue, patients often struggle with adherence to these interventions in clinical practice. Game-based eHealth interventions are believed to address adherence issues by making the intervention more accessible and engaging.

**Objective:**

This study aims to compile empirical evidence on game-based eHealth interventions for fatigue in individuals with chronic diseases and to evaluate their effectiveness in alleviating fatigue.

**Methods:**

A comprehensive literature search was performed across Embase, MEDLINE ALL, PsycINFO, Web of Science Core Collection, Cochrane Central Register of Controlled Trials, and Google Scholar in August 2021. Study characteristics and outcomes from the included studies were extracted, and a random-effects meta-analysis was conducted. Sensitivity and subgroup analyses were performed to identify sources of heterogeneity.

**Results:**

Of 1742 studies identified, 17 were included in the meta-analysis. These studies covered 5 different chronic diseases: multiple sclerosis (n=10), cancer (n=3), renal disease (n=2), stroke (n=1), and Parkinson disease (n=1). All but 1 study used exergaming interventions. The meta-analysis revealed a significant moderate effect size in reducing fatigue favoring the experimental interventions (standardized mean difference [SMD] –0.65, 95% CI –1.09 to –0.21, *P*=.003) compared with control conditions consisting of conventional care and no care. However, heterogeneity was high (I2=85.87%). Subgroup analyses were conducted for the 2 most prevalent diseases. The effect size for the multiple sclerosis subgroup showed a trend in favor of eHealth interventions (SMD –0.47, 95% CI –0.95 to 0.01, *P*=.05, I2=63.10%), but was not significant for the cancer group (SMD 0.61, 95% CI –0.36 to 1.58, *P*=.22). Balance exercises appeared particularly effective in reducing fatigue (SMD –1.19, 95% CI –1.95 to –0.42, *P*=.002).

**Conclusions:**

Game-based eHealth interventions appear effective in reducing fatigue in individuals with chronic diseases. Further research is needed to reinforce these findings and explore their impact on specific diseases. Additionally, there is a lack of investigation into interventions beyond exergaming within the field of game-based learning.

## Introduction

Chronic diseases are a major cause of morbidity worldwide, with their prevalence steadily increasing due to a growing and aging population, improved disease detection, and advancements in medical treatments, leading to greater longevity [1,2]. Chronic diseases are conditions that persist over a long period or recur frequently, often requiring ongoing medical attention [3,4]. The management of chronic diseases is shifting from cure to care and prevention strategies, with a particular focus on lifestyle management [5]. In addition to the impact of the chronic disease itself, several commonly associated symptoms—such as depression, anxiety, and fatigue—affect quality of life and should be included in routine care [6-8].

Fatigue is one of the most prevalent of these symptoms [8]. It is defined as an overwhelming sense of tiredness and exhaustion that arises without provocation and cannot be relieved by rest [9,10]. Connolly et al [8] found that patients often report fatigue as one of the most debilitating symptoms, significantly impacting daily functioning and quality of life. They report that fatigue occurs across a range of chronic diseases, including multiple sclerosis (MS) and cancer. A recent meta-analysis evaluated the prevalence of severe and chronic fatigue in a cohort of individuals with chronic diseases, finding that 23% experienced severe fatigue and 17% suffered from chronic fatigue [11].

Over the past decades, nonpharmacological treatments for fatigue have been increasingly developed and investigated. Meta-analyses indicate that physical exercise can reduce fatigue severity across various chronic diseases [12-17]. Other successful interventions include psychological therapies—such as cognitive-behavioral therapy, psychoeducation, or mindfulness—whether as standalone treatments or in combination with exercise, as well as relaxation therapies [18,19].

Despite these findings, patients often struggle with adhering to interventions [20,21]. Evidence indicates that the reasons for nonadherence are diverse, including barriers such as time, costs, location, comorbidities, and particularly a lack of motivation [21-25]. To be successful, interventions should be designed to address and overcome these barriers to adherence. This is the goal of game-based interventions, which aim to make treatment easily accessible and highly engaging. This is one reason why such interventions have become increasingly popular in recent years. Games are known to enhance motivation, attention, and learning, among other benefits [26]. Game-based interventions leverage these benefits by embedding therapeutic goals within a game (serious gaming). Evidence indicates that these interventions can significantly improve treatment adherence in chronic conditions compared with standard care [27]. Additionally, from a financial perspective, game-based interventions are attractive to health care providers and insurance companies due to their cost-effectiveness [28-30].

Several studies have investigated the effects of game-based eHealth interventions in individuals with chronic diseases, yielding promising results across a range of outcomes. These interventions include exergames (ie, game-based exercise programs [31]), virtual reality (VR) tools [32], and serious game applications [33]. For example, Kato et al [34] investigated the effect of a serious game designed to improve adherence and other behavioral outcomes in children with cancer, finding that it successfully enhanced medication adherence and self-efficacy in the target group. In a study by Del Corral et al [35], exergaming was found to lead to significantly greater improvements in exercise capacity, muscular strength, and quality of life in children with cystic fibrosis compared with the control group receiving conventional care.

With the accumulation of numerous studies over the past decade, evidence in this field has been synthesized in meta-analyses. Rutkowski et al [36] found that VR interventions appear to be effective in alleviating fatigue in individuals with cancer. Cugusi et al [37] reported small but significant effect sizes for improving health-related quality of life with experimental exergaming interventions in people with various chronic diseases, including Parkinson disease, Alzheimer disease, and stroke. Seiler et al [38] also found promising effects of various types of eHealth interventions in reducing fatigue in individuals with cancer.

However, to date, no meta-analysis has investigated the effects of (1) different game-based eHealth interventions on (2) the reduction of fatigue in (3) individuals with various chronic conditions.

This paper aims to fill this gap by systematically aggregating the findings from these studies to assess the effectiveness of game-based eHealth interventions in alleviating fatigue. The goal is to determine whether these interventions can serve as a suitable alternative to conventional treatments.

## Methods

### Selection Criteria

We included randomized and nonrandomized controlled trials that reported the effects of video game interventions on fatigue in individuals with chronic diseases. For this study, we defined a video game as a digital or electronic game where players interact with the game by manipulating images on a video screen. A “game” was defined as an engaging, amusing, and structured form of play conducted according to a set of rules with the aim of achieving a specific objective. Chronic diseases are defined as conditions that persist or recur over an extended period and require ongoing medical attention or limit activities of daily living [3,4]. We focused on pathological fatigue, defined as physical, emotional, or mental tiredness/exhaustion related to chronic disease or its treatment [39]. This type of fatigue is characterized by its prolonged, severe, progressive nature or its occurrence without provocation. For practical reasons, we included only journal articles published in English. All studies had to include a T1 measure with a measure of change from the baseline and a control group from the same disease population receiving a different or no intervention.

We excluded trials involving healthy volunteers, individuals with acute diseases, and those with fibromyalgia. The clinical population with fibromyalgia was excluded due to its high heterogeneity, unclear etiology, and purely clinical diagnosis, as there are no specific laboratory abnormalities associated with it [40]. Therefore, fibromyalgia is unsuitable for this meta-analysis due to its heterogeneous nature and unclear etiology, making it difficult to detect the group effects of an intervention. Articles focusing on different types of fatigue, such as fatigue after exertion or transient fatigue, were also excluded. Additionally, we excluded reviews, descriptive and observational studies, study protocols, case studies, uncontrolled studies, conference abstracts, trial registries, posters, and books, as well as studies that used nonstandardized measuring scales for fatigue.

### Search Strategy

A medical information specialist from the Erasmus MC Medical Library conducted a comprehensive literature search on August 25, 2021. To ensure the findings were up-to-date, a second search was carried out on March 2, 2023. Both searches utilized the following databases: Embase, MEDLINE ALL, PsycINFO, Web of Science Core Collection, Cochrane Central Register of Controlled Trials, and Google Scholar. For both searches, the coverage years varied by database (Multimedia Appendix 1). Nonetheless, the majority of articles were published within the last 3 decades.

The search terms “game,” “video,” “fatigue,” and related keywords were used. A separate search strategy was developed for each database (Multimedia Appendix 1). We did not include “chronic disease” or specific diseases as search terms, as we deemed the risk of missing relevant studies due to incomplete disease terms to be too high.

### Selection Procedure

After removing duplicates, the titles and abstracts were screened by 2 of the authors (LSW and JSL). The full-text papers were then extracted and screened by the same authors along with an additional author (BD). The selection of articles was compared among the authors at all stages of the process. In cases of disagreement, the articles were discussed until a consensus was reached. The authors of the papers were contacted by email when relevant information was missing or inconsistent. Articles were excluded if the authors did not respond.

### Assessment of Study Quality

For each study, the risk of bias was assessed by the author LSW using the risk of bias 2 tool (Cochrane Risk of Bias Tool for Randomized Trials) [41]. The assessment covered the following categories: randomization process, deviations from intended interventions, missing outcome data, measurement of the outcome, and selection of reported results. Based on the assessment of the individual categories, each study was classified into 1 of 3 overall risk of bias levels: “low risk of bias,” “some concerns,” or “high risk of bias.” The overall classification was determined by the lowest rating among the individual categories (eg, if 4 of the 5 categories were rated “low risk of bias” but 1 was rated “high risk of bias,” the overall classification would be “high risk of bias”).

### Data Extraction

After the selection process, one author (LSW) performed data extraction for each article. The extracted data included diagnosis, author, year of publication, sample size, mean age, percentage of female participants, interventions in both the experimental and control groups, duration of the interventions in weeks, and key findings. All data were entered into Comprehensive Meta-Analysis (CMA) software version 2 (Biostat, Inc.).

### Synthesis of Results

The analyses were conducted using CMA [42]. For our outcome variable, fatigue, the mean scores and SDs for pre- and postintervention (ie, baseline and T1) were either extracted directly from the articles or calculated from median scores and IQRs using the formula described by Wan et al [43]. We followed the guidelines in the Cochrane Handbook for Systematic Reviews of Interventions to calculate the pre-post correlation [44]. It was calculated directly for studies where the SD of change from baseline to T1 was available. For other studies, we imputed the correlation by averaging the calculated pre-post correlations. Additionally, we entered data on sample size per condition, diagnosis, assessment instrument (Visual Analog Scale [VAS] vs questionnaire), mean age, percentage of female participants, modality and type of intervention (nonimmersive, immersive VR, non–VR game; balance, fitness, cognition), intervention duration in weeks, type of control intervention (no care vs conventional care), setting (hospital vs home), supervision, and, where possible, disease severity into the CMA worksheet. Two of the included studies were crossover randomized controlled trials (the remainder were parallel randomized controlled trials). For 1 of the crossover studies, we used only the T1 measure for comparison, which included data only from the period before the crossover [45]. For the other crossover study, we used data from both periods combined (ie, before and after the crossover) because only these combined data were available [46]. In studies measuring different dimensions of fatigue, the dimension reporting the average fatigue measure was used. In studies with 2 control groups—1 receiving a conventional intervention and 1 with no intervention—we chose the inactive control group. In a study comparing 2 interventions using different VR systems with a control group, the aggregated mean of the 2 experimental conditions was used [47]. For studies with 3 measurement points, data from the measures immediately before and after the intervention were utilized.

**Figure 1:**
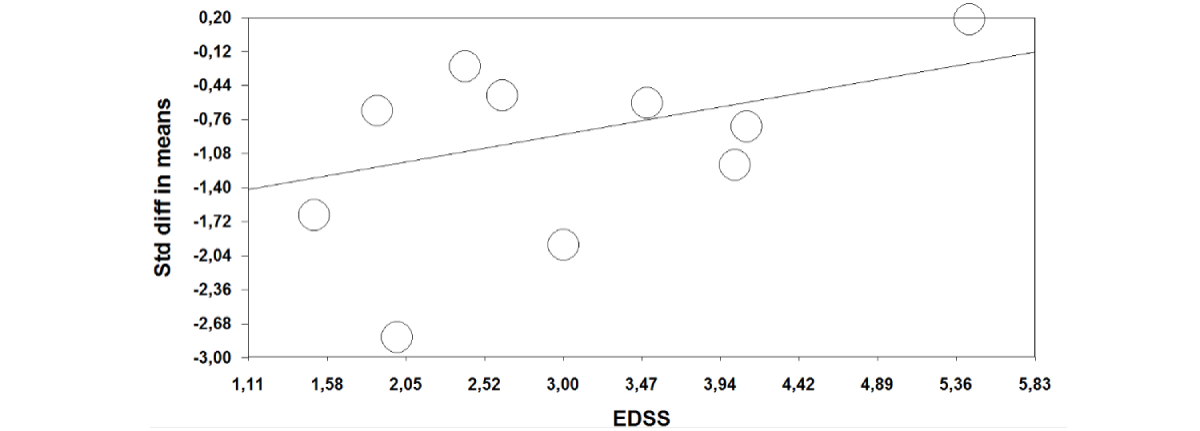
Scatterplot showing the meta-regression of all multiple sclerosis studies with the Expanded Disability Status Scale (EDSS) as the predictor variable.

First, effect sizes were calculated as standardized mean differences (SMDs) to account for possible differences in measurement scales. We conducted a meta-analysis to determine the overall effect sizes for the experimental condition compared with the control condition using a random-effects model. The more conservative random-effects model was chosen over the fixed-effects model due to the expected heterogeneity among studies and because random-effects models are recommended for analyzing data collected in real-world settings rather than controlled laboratory environments [48]. Heterogeneity was estimated using the *I*^2^ index, which describes the percentage of variation attributable to study heterogeneity rather than chance [49], with ≥75% indicating considerable heterogeneity. To explore sources of heterogeneity, we performed sensitivity analyses (by excluding low-quality studies and outliers), moderator analyses, and meta-regressions. Low-quality studies were defined as those with a high risk of bias, as identified by the risk of bias assessment. Outliers were defined as studies where the 95% CI did not overlap with the 95% CI of the pooled effect size. For the moderator analyses, studies were grouped by diagnosis, age, and type of experimental and control interventions, provided there was more than 1 study per group. Additionally, we conducted analyses excluding studies using VAS, exploring the impact of supervision, and distinguishing between studies conducted at home versus those conducted in a hospital setting. A random-effects meta-regression using the method of moments was conducted with gender and disease duration as predictors. For studies on MS, we additionally performed a meta-regression with disease severity, which was measured using the Expanded Disability Status Scale (EDSS) [50], as a predictor. This is shown in Figure 1.

Finally, to check for publication bias, we generated a funnel plot by plotting the SMD against the SE of all studies and assessed it for asymmetry. Additionally, we quantified potential publication bias statistically using the Egger test of the intercept [51].

## Results

### Study Selection

**Figure 2:**
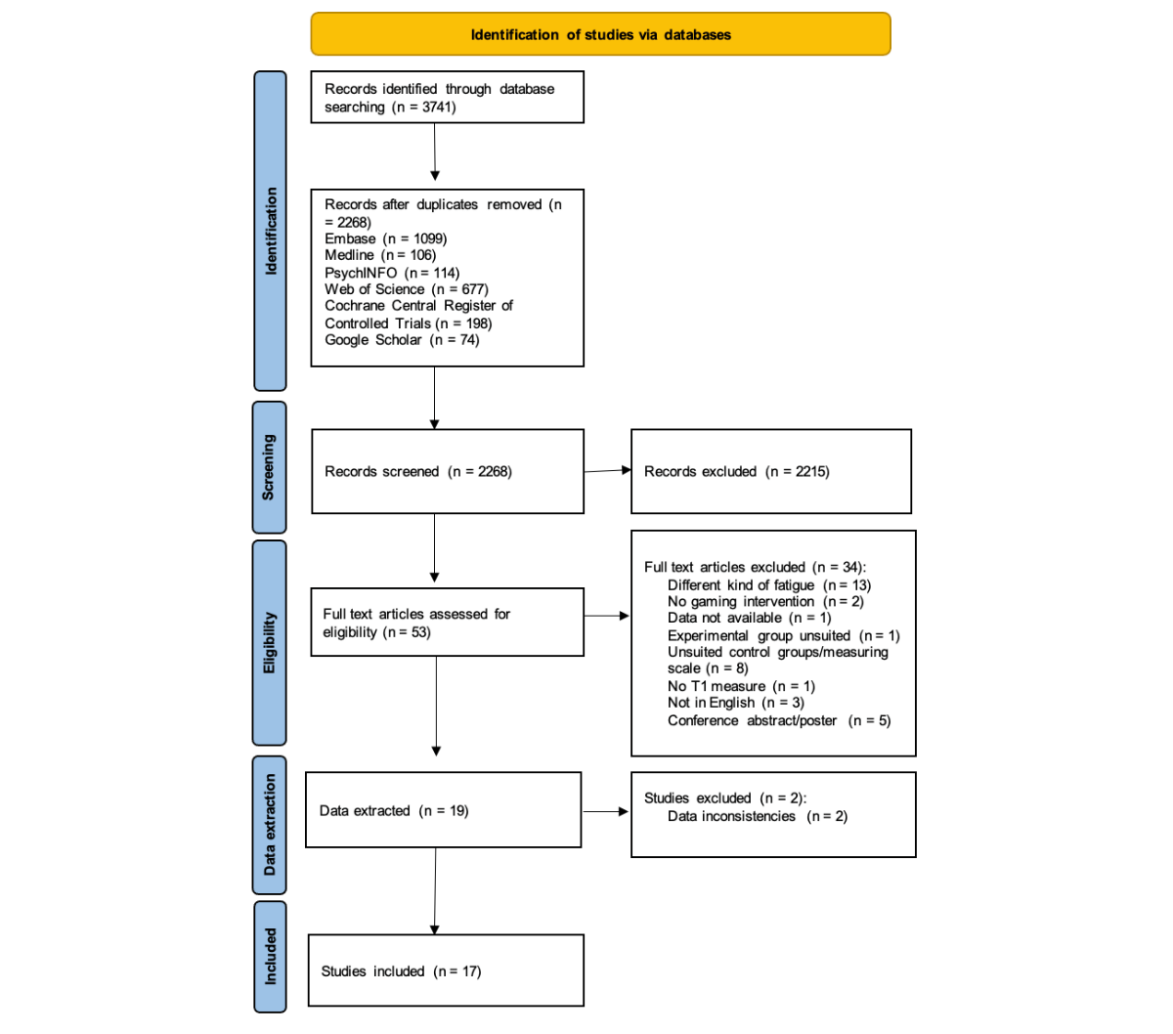
Preferred Reporting Items for Systematic Reviews (PRISMA) flowchart-diagram for study selection.

A total of 3741 articles were identified through the literature search, of which 2268 remained after duplicate removal. An overview of the selection process is shown in Figure 2. After screening titles and abstracts, and discussing differences in opinion between the authors (for 14 articles), a total of 53 articles were selected for full-text screening. After independently screening the full text of these studies, the authors discussed discrepancies in study selection for 8 studies until a consensus was reached. Authors were contacted for missing information in 2 cases. We received a reply for 1 article, which led to its exclusion. The other study was excluded due to the lack of a response. In total, 19 articles were excluded after full-text review. Most were excluded because the fatigue measure pertained to exertion from the intervention itself. Relevant data were then extracted from the remaining 19 studies. During this process, 2 additional studies were excluded due to data inconsistencies; we contacted the authors but did not receive a reply. This left us with a total of 17 studies included in the data synthesis for the meta-analysis.

### Characteristics of the Included Studies

Table 1 presents the characteristics of the included studies, organized by participant diagnosis: MS (n=10) [45,47,52-59], cancer (n=3) [46,60,61], renal disease (n=2) [62,63], Parkinson disease (n=1) [64], and poststroke (n=1) [65]. The studies were published between 2013 and 2023 and were all randomized controlled trials, including 2 crossover trials [45,46] and 15 parallel trials [47,52-65]. The number of participants ranged from 20 to 52. The mean age of participants ranged from 7.9 to 68.7 years, with only 1 study [60] including children. For the 16 studies that included adults, the age range was 32.3-68.7 years (median 45 years) [45-47,52-59,61-65]. The mean percentage of female participants ranged from 0% (0/42) to 90% (38/42; median 61.50%). Sixteen studies used VR exergames, which included either balance exercises (n=5) [47,52,55,57,65] or fitness exercises (n=11) [45,46,53,56,58-64]. One study used a serious Nintendo DS game designed to train cognitive functions such as working memory, spatial recognition, processing speed, and mental reasoning in healthy individuals [54]. Thus, the term “game-based eHealth interventions” is technically too broad. Throughout this paper, we used this term to include the serious gaming study as well. The VR technology varied across studies, with most using nonimmersive VR systems (n=14) [45-47,52,53,55,56,58-64]. The control interventions also exhibited some heterogeneity across studies. We categorized the control interventions into 2 groups: the conventional training group (n=7) [46,52,53,55,59,64,65], which involved traditional nonvideo game interventions targeting the same abilities as the experimental intervention, and the no-exercise control group (n=10) [45,47,54,56-58,60-63], which involved no specific intervention beyond the normal level of activity. The duration of the interventions varied significantly between studies, ranging from 2 to 24 weeks (median 8 weeks). Fatigue was measured using various questionnaires, with 1 study using a VAS to assess fatigue severity [62].

**Table 1:** Study characteristics and key findings of the included studies that reported on the effect of game-based eHealth interventions on the reduction of fatigue in people with chronic diseases. Studies are sorted according to diagnosis.

Diagnosis and study		Participants			Intervention				Key findings
		N	Age (years), mean	Female, n/N (%)	Experimental	Control	Duration (weeks)		
**Multiple sclerosis**									
	Brichetto et al [52]	36	42	22/36 (61)	Balance, nonimmersive VR^a^	Conventional training	12	Significant decrease in fatigue (MFIS^b^) after treatment compared with the baseline and control groups	
	Cuesta-Gómez et al [53]	30	46.3	18/30 (60)	Fitness, nonimmersive VR	Conventional training	10	No significant decrease in fatigue after treatment (FSS^c^)	
	De Giglio et al [54]	35	43.8	26/35 (74)	Cognition, Gameboy	No exercise	8	No significant decrease in fatigue after treatment (MFIS)	
	Khalil et al [55]	32	37.4	22/32 (69)	Balance, nonimmersive VR	Conventional training	6	Significant decrease in fatigue (MFIS) after treatment compared with the control group	
	Ozdogar et al [56]	59	40.2	43/59 (73)	Fitness, nonimmersive VR	No exercise	8	No significant decrease in fatigue in the experimental and control groups (MFIS)	
	Ozdogar et al [59]	30	37.6	21/31 (68)	Fitness, nonimmersive VR	Conventional rehabilitation	6	Significant decrease in fatigue (MFIS) in the experimental group compared with baseline and the control group	
	Ozdogar et al [58]^d^	31	40.3	20/31 (65)	Fitness, nonimmersive VR	No exercise	8	Significant increase in sleep quality in the experimental group compared with baseline and the control group	
	Ozdogar et al [58]^e^	34	40.8	21/34 (62)	Fitness, nonimmersive VR	No exercise	8	No significant increase in sleep quality in the experimental and control groups	
	Ozkul et al [57]^e^	39	32.3	30/39 (77)	Balance, immersive VR	No exercise	8	Significant decrease in fatigue (FSS) after treatment compared with baseline and the control group	
	Thomas et al [45]^e^	29	49.3	27/30 (90)	Fitness, nonimmersive VR	No exercise	24	Increased fatigue after treatment in both experimental and control groups (FSI^f^)	
	Yazgan et al [47]^e^	42	43.7	38/42 (90)	Balance, nonimmersive VR	No exercise	8	Significant decrease in fatigue (FSS) after treatment compared with baseline and the control group	
**Cancer**									
	Hamari et al [60]	36	7.8	10/36 (28)	Fitness, nonimmersive VR	No exercise	8	Change in fatigue was similar in both groups (PedsQL^g^)	
	Kobayashi et al [46]	22	44.8	4/22 (18)	Fitness, nonimmersive VR	Conventional training	2	Increase in fatigue after the intervention in the experimental group and a significant decrease in the control group (POMS-sf Fatigue^h^)	
	Villumsen et al [61]	46	68.7	0/46 (0)	Fitness, nonimmersive VR	No exercise	12	No significant decrease and no significant difference in change in fatigue between groups (FACT-F^i^)	
**Renal disease**									
	Cho and Sohng [62]	48	59.3	20/48 (42)	Fitness, nonimmersive VR	No exercise	8	Significant decrease in fatigue in the experimental group but not in the control group (VAS^j^)	
	Chou et al [63]	64	59.3	28/64 (44)	Fitness, nonimmersive VR	No exercise	4	Significant decrease in fatigue in both groups (NFSHD^k^); no significant difference between groups	
**Parkinson disease**									
	Ribas et al [64]	20	61	8/20 (40)	Fitness, nonimmersive VR	Conventional training	12	Significant decrease in fatigue in the experimental group but not in the control group (FSS)	
**Stroke**									
	de Rooij et al [65]	52	63	16/52 (31)	Balance, immersive VR	Conventional training	6	No significant decrease and no significant difference in change of fatigue between groups (FSS)	

^a^VR: virtual reality.

^b^MFIS: Modified Fatigue Impact Scale.

^c^FSS: Fatigue Severity Scale.

^d^With restless legs syndrome.

^e^Without restless legs syndrome.

^f^FSI: Fatigue Symptom Inventory.

^g^PedsQL: Pediatric Quality of Life Inventory.

^h^POMS-sf: Profile of Mood States—short form.

^i^FACT-F: Functional Assessment of Cancer Therapy—Fatigue.

^j^VAS: Visual Analog Scale.

^k^NFSHD: Novel Fatigue Scale for Hemodialysis.

### Risk of Bias of Studies

**Figure 3:**
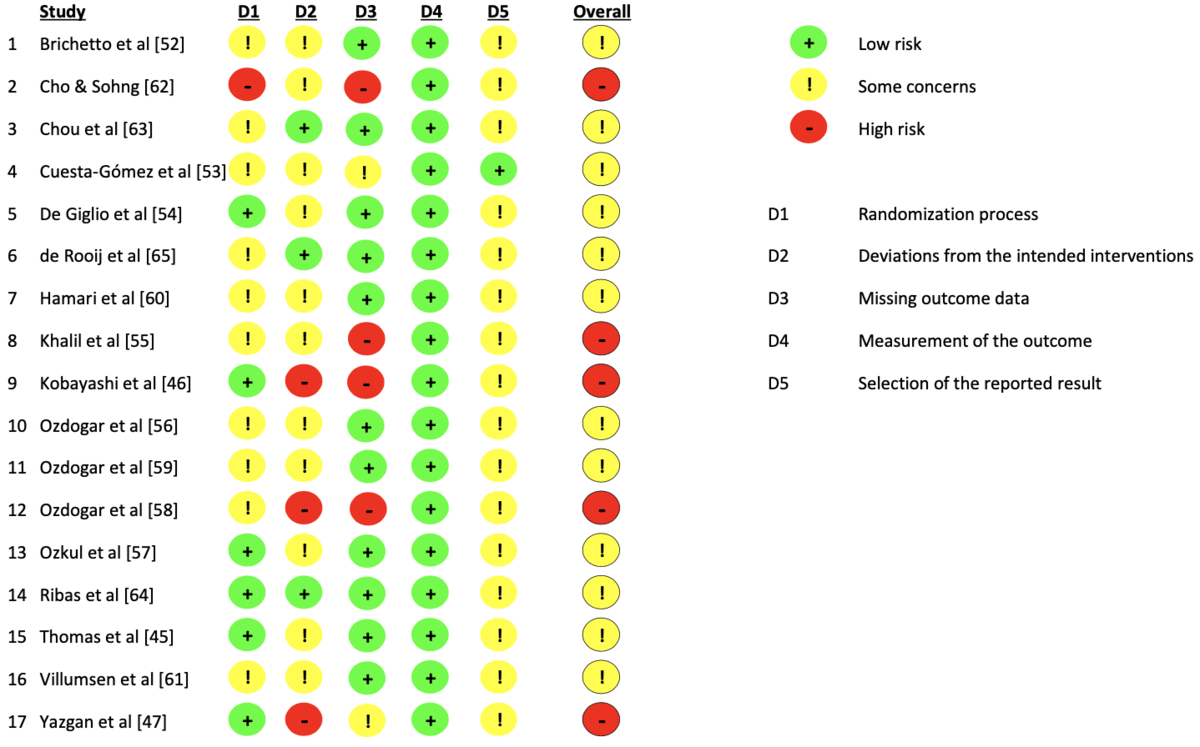
Risk of bias assessment for all included studies.

Study quality was low or moderate in all studies (n=5 and n=12, respectively), with none rated as high quality (Figure 3). The primary reasons for low-quality ratings were issues with the randomization process and handling of missing data.

While all studies were randomized, 2 utilized a cluster-randomization procedure, with treatment allocation based on either the days participants visited the hospital [62] or the hospital wards to which they were assigned [63]. For Chou et al [63], we can assume that participant allocation was concealed from the investigator; however, this was not clear for Cho and Sohng [62]. For the other studies, the randomization process was truly random. However, there was some concern about the risk of bias in 9 of the 17 (53%) studies [52,53,55,56,59-61,63,65] due to missing or doubtful information about allocation concealment.

In 10 of the 17 (59%) studies [45-47,53-57,60,62], there was concern about the risk of bias because the authors did not implement an intention-to-treat analysis to account for missing or lost data. However, the missing data were either balanced across studies or not substantial enough to significantly impact the results. By contrast, 2 (12%) studies [46,47] exhibited a high risk of bias due to substantial issues with missing data.

In 8 of the 17 (47%) studies [46,47,53,55,57,58,60,62], it could not be ruled out that the missing data were related to the outcome itself (ie, fatigue), potentially influencing the overall results. Possible reasons related to the outcome were lack of motivation or excessive fatigue preventing participation. Issues related to participants’ schedules or travel time were judged as unrelated to the outcome. In 4 of these studies [46,55,58,62], a high risk of bias was concluded as it was likely that the missing data depended on the true value of the outcome. In the other 4 studies [47,53,57,60], there were some concerns about the risk of bias, but the proportion of missing data and reasons for it were balanced across groups.

The risk of bias in the measurement of outcomes was low across all studies. However, there was some concern regarding the selection procedure of reported results in 16 of the 17 (94%) studies [45-47,52-61,63-65]; specifically, 16 (94%) studies did not indicate whether the analysis followed a prespecified plan [45-47,52,54-65]. Additionally, 4 (24%) studies began before the trial had been preregistered.

### Main Analyses

**Figure 4:**
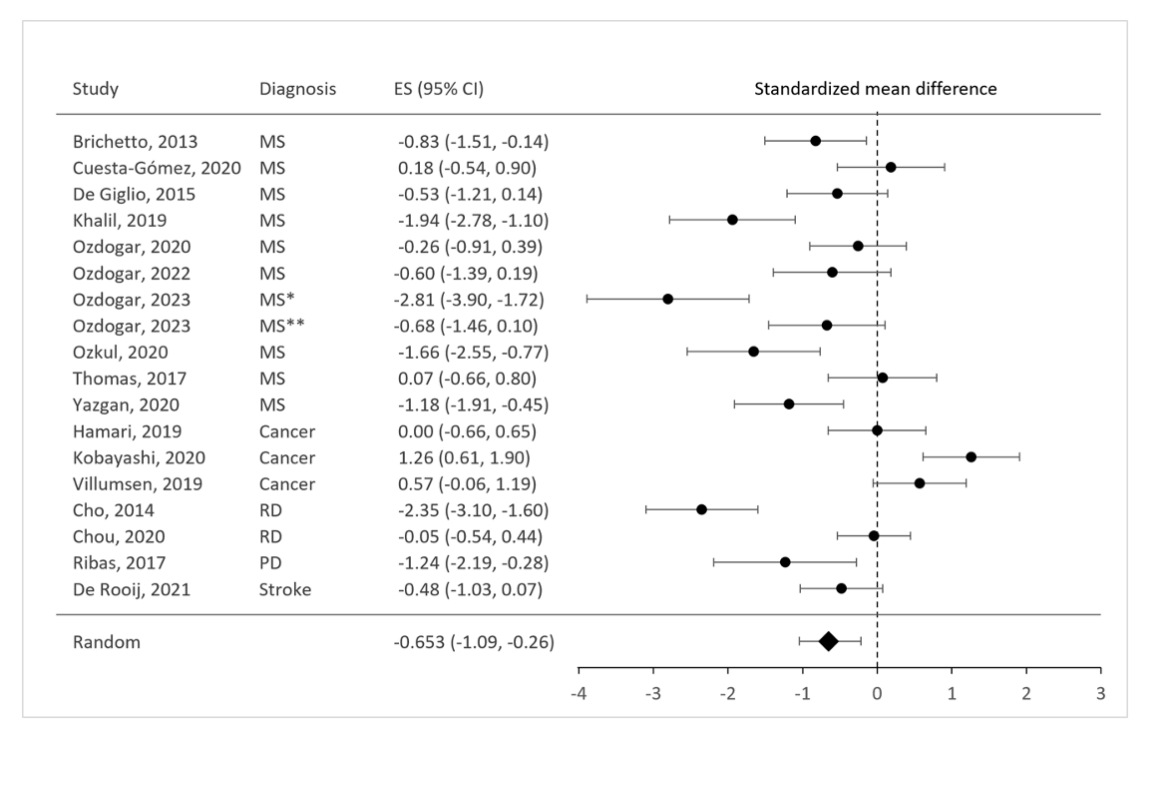
Random-effect meta-analysis for the effect of serious gaming on fatigue. ES: effect size; MS: multiple sclerosis; PD: Parkinson disease; RD: renal disease. *With restless leg syndrome; **without restless leg syndrome.

It was possible to calculate effect sizes for fatigue reduction across all studies. Figure 4 presents the SMD and 95% CIs for each study. A negative effect size indicates that the experimental intervention was more effective in reducing fatigue compared with the control intervention. In 4 studies [45,46,53,61], the effect sizes were positive, meaning that the control intervention was more effective in reducing fatigue than the experimental intervention. Three studies were identified as outliers, 2 [46,62] of which were also rated as low quality. The third outlier was the study by Villumsen et al [61], which also reported no reduction in fatigue following the intervention. This lack of effect may be related to the study’s population, which consisted entirely of males with a mean age of 68.7 years, the oldest among all the studies. The authors suggest that the lack of supervision over the exercise intensity in the home-based intervention might explain the findings. The overall effect size, calculated using a random-effects model, indicated a significant moderate effect of video game interventions on fatigue reduction compared with control interventions (SMD –0.65, 95% CI –1.09 to –0.21, *P*=.003). However, there was considerable heterogeneity (*I*^2^=85.87%). To investigate the sources of this heterogeneity, several additional analyses were conducted.

First, we performed sensitivity analyses by excluding the low-quality and outlier studies. This included 4 studies [46,47,55,62] with a high risk of bias and 1 additional outlier [61], leaving us with 10 studies. Despite this more rigorous analysis, the effect size remained significant (SMD –0.42, 95% CI –0.74 to –0.10, *P*=.01). Although heterogeneity was reduced, it remained substantial (*I*^2^=54.88%). When the study using a VAS was excluded (n=1) [62], the effect size was smaller but still significant (SMD –0.55, 95% CI –0.95 to –0.14, *P*=.009).

Second, we performed moderator analyses with diagnosis, type of intervention, type of control condition, and age as moderators for all groups where data from more than 1 study were available. Grouping studies according to diagnosis revealed a large and significant effect size for MS (SMD –0.87, 95% CI –1.34 to –0.41, *P*<.001, n=10). After removing low-quality and outlier studies, the effect size decreased to a trend (SMD –0.47, 95% CI –0.95 to 0.01, *P*=.05, *I*^2^=63.10%, n=6). For cancer, the pooled effect size was positive, indicating that the control intervention was more effective than the experimental intervention, but this effect size was not statistically significant (SMD 0.61, 95% CI –0.36 to 1.58, *P*=.22, n=3). For both MS and cancer, heterogeneity was reduced but remained substantial (*I*^2^=77.9% for MS and *I*^2^=72.33% for cancer). For renal disease, with only 2 studies available [62,63], the pooled effect size was –1.13 (95% CI –2.32 to 0.05, *P*=.06), and heterogeneity was notably high (*I*^2^=96.08%).

Interventions involving balance exercises (n=5) showed a large effect size of –1.19 (95% CI –1.95 to –0.42, *P*=.002). By contrast, for fitness interventions (n=10), the effect size was nonsignificant (SMD –0.44, 95% CI –1.02 to 0.13, *P*=.20). Heterogeneity remained substantial for both categories (*I*^2^=62.78% for balance exercises and *I*^2^=87.15% for fitness interventions). We could not pool effects for cognitive interventions, as only 1 study investigated this category [54]. Sensitivity analysis, which excluded low-quality and outlier studies, confirmed these results while substantially reducing heterogeneity (*I*^2^=58.83% with n=3 for the balance group and *I*^2^=23.60% with n=6 for the fitness group). These findings suggest that game-based balance exercises, in particular, are effective interventions for reducing fatigue in individuals with chronic diseases.

Grouping studies by the type of control group used revealed nonsignificant effect sizes for the conventional training control groups (SMD –0.49, 95% CI –1.12 to 0.15, *P*=.20, *I*^2^=86.7%, n=7), but significant effect sizes for the no-exercise control groups (SMD –0.75, 95% CI –1.33 to –0.18, *P*=.01, *I*^2^=85.6%, n=10).

Significant differences were found when comparing participants with a mean age below 55 years (SMD –0.65, 95% CI –1.16 to –0.13, *P*=.02, *I*^2^=84%, n=12), but no significant difference was observed for those above 55 years (SMD –0.68, 95% CI –1.59 to 0.23, *P*=.15, *I*^2^=90%, n=5).

Third, we conducted a meta-regression using the method of moments in a random-effects model to estimate the effect of gender and duration of intervention on the impact of game-based interventions. Neither gender nor duration significantly influenced the effect size for fatigue reduction (*P*=.08 and *P*=.86, respectively). However, the presence of supervision and the location of the studies have significantly influenced the effect size. For both factors, a meta-regression was conducted using a random-effects model. Supervision significantly affected the effect size, with supervised interventions showing a significant effect (SMD –0.86, 95% CI –1.39 to –0.33, *P*=.001), whereas interventions without supervision showed no significant effect (SMD 0.04, 95% CI –0.42 to 0.49, *P*=.88). The meta-regression also revealed a significant effect for studies conducted in a hospital setting (SMD –0.79, 95% CI –1.30 to –0.30, *P*=.002), contrasting with those conducted at home, which showed no significant effect (SMD 0.04, 95% CI –0.61 to 0.69, *P*=.90).

We performed an additional meta-regression with disease severity as a predictor for all the MS studies where it was reported (n=9; not reported in n=1) [45,47,52-59]. Increased severity was associated with a smaller effect of the intervention (SMD –0.27, 95% CI 0.06-0.48, *P*=.01). When high risk of bias studies were excluded, the association remained, although slightly weaker (SMD 0.25, 95% CI 0.02-0.49, *P*=.04, n=5) [46,47,55,58,62].

### Publication Bias

The funnel plots for all included studies and the MS subgroup displayed asymmetry [45,47,52-59]. The Egger test of the intercept confirmed that this asymmetry was statistically significant, with *P* values of .006 and .004, respectively, indicating evidence of publication bias. This suggests an overrepresentation of studies with positive results, which should be considered when interpreting the findings. However, after excluding studies with a high risk of bias, the observed asymmetry was no longer statistically significant (*P*=.25) [46,47,55,58,62].

## Discussion

### Principal Findings

In recent years, the digitalization and gamification of interventions have garnered increasing attention as alternatives or complements to conventional treatment approaches. This paper aimed to evaluate the efficacy of game-based eHealth interventions in reducing fatigue among individuals with chronic diseases. We included 17 randomized controlled trials published between 2013 and 2023, encompassing 5 different types of chronic diseases. The relative recency of publications and the small number of studies illustrate that the field of (game-based) eHealth is still in its infancy. The types of interventions were fairly homogeneous, with all but 1 study focusing on exergaming interventions [54]. The remaining studies evaluated a serious game aimed at improving cognition. This trend, although on a smaller scale, mirrors the evidence base for conventional rehabilitation approaches for fatigue, where the majority of studies also focus on physical exercise interventions. However, given the positive findings for psychological interventions, particularly when combined with exercise interventions [18], future eHealth interventions should also explore these approaches.

Findings from this meta-analysis suggest that current game-based eHealth interventions may effectively reduce fatigue in people with chronic diseases. With a moderate effect size, these interventions could potentially be more effective for fatigue compared with other treatment goals, such as knowledge and self-management [66], self-efficacy [67], and health-related quality of life [37], where previous meta-analyses reported smaller effect sizes. Additionally, they appear to be as effective as many conventional (non–game-based) interventions, which typically report moderate effect sizes [18]. Some meta-analyses investigating the effect of exercise therapy on disease-related fatigue (such as in cancer and chronic obstructive pulmonary disease) reported larger effect sizes for physical exercise therapies [13,15], a finding also supported by individual studies in this meta-analysis [47,52,55,57,62,64]. The comparable effectiveness of game-based eHealth interventions is crucial for them to become a viable alternative to conventional interventions. Thus, this result represents an important first step in exploring the potential of game-based eHealth interventions.

With regard to individual chronic diseases, findings from this meta-analysis were less straightforward. Game-based interventions appear effective for MS, but not for cancer. Cancer is a heterogeneous disease with variable cancer-related fatigue. The underlying pathophysiology is relatively well investigated and is likely multifactorial, involving inflammation, disruptions in the hypothalamic–pituitary–adrenal axis, and activation of the autonomic nervous system [68,69]. However, it is influenced by several factors including the type of cancer, the stage of the disease, and the treatment—all of which varied across and within the study populations of the included cancer studies. The considerable heterogeneity of the cancer group, including a wide mean age range from 8 to 69 years, might explain the lack of a treatment effect in this group. By contrast, MS typically presents with a more homogeneous course, commonly consisting of exacerbations and stable phases [70], and all of the MS studies included here focused on patients in a stable phase. As the onset of MS typically occurs between 20 and 40 years of age [71], the study population for MS was more homogeneous in terms of age, ranging from 31 to 49 years. Besides disease-related differences, variations in results might be attributed to statistical power issues due to the limited number of studies [72]. This limitation increases the likelihood of fluctuations due to chance, particularly in the cancer group, where 1 study was an outlier [61] and another was of low quality [46]. Interestingly, 1 of the cancer studies [46] found that the experimental group experienced an increase in fatigue after the intervention. The authors suggested that this might be due to an inappropriate exercise load, as patients were unable to adjust it according to their needs in the experimental condition. Additionally, “fatigue” might have been interpreted as “exercise load,” given that the Profile of Mood States—short form (POMS-sf) measuring scale used in the study assesses “general fatigue” nonspecifically.

Another striking finding of this meta-analysis was the clear difference between balance exercises and fitness exercises. The balance exercises showed a markedly larger effect size in reducing fatigue compared with fitness exercises (SMD –1.19 vs SMD –0.17). It is important to note that the balance exercise group was more homogeneous in terms of patient diagnoses, with only 1 study including patients after stroke and the rest consisting of patients with MS [65]. The fitness group included patients with 4 different diagnoses, which might contribute to the observed heterogeneity and complicate the comparison with the balance group. However, a similar observation is evident within the MS studies: all 4 balance studies favored the experimental intervention [47,52,55,57], whereas only 3 [57-59] of the 6 fitness studies did [45,53,56]. These findings contrast with a recent randomized controlled trial by Callesen et al [73], which reported conventional balance training and exercise training as equally effective in reducing fatigue among patients with MS. However, our results align with Hebert and Corboy [74], who demonstrated a significant relationship between fatigue and balance in patients with MS. Additionally, evidence from healthy participants suggests that balance exercises not only improve balance but also muscle strength [74,75]. This dual benefit might make balance exercises more effective than pure strength exercises in reducing fatigue, as they address both balance and strength—factors associated with fatigue. Additionally, balance exercises might be more enjoyable and less demanding than fitness exercises. It is also worth noting that 2 studies in the balance group incorporated additional interventions: 1 included walking alongside balance exercises [57] and another combined Pilates with balance exercises [65]. Given the small number of studies in the balance group (n=5), the effects of these 2 studies significantly influence the overall effect size for this group. Overall, the observation that balance exercises appear particularly effective in reducing fatigue is intriguing and warrants further investigation in future research. It also underscores the importance of developing tailored treatment programs for fatigue, as the underlying mechanisms may vary between different diseases [76].

In this meta-analysis, only 4 studies utilized tailored interventions specifically designed for rehabilitation [53-55,65], while the remaining 13 studies used off-the-shelf commercial games [45-47,52,56-64]. According to serious game design theory, considering the unique interests and needs of the target group leads to the best outcomes [77,78]. Nonetheless, 6 [47,52,60,62-64] out of the 11 studies using commercial games were successful in alleviating fatigue [45-47,52,54,56,60-64]. Gender did not appear to influence the effectiveness of the interventions, which contrasts with the assumption of game design theory. This suggests that the success of commercial games might stem from their broad appeal, as developers aim to meet the needs of diverse target groups to maximize their reach. Yet again, age, particularly a mean age below 55 years, had a significant effect on the effectiveness of the intervention. The literature presents mixed findings regarding the influence of age and gender on treatment outcomes in eHealth interventions. Some studies report differences attributed to these variables [79,80], while others do not [81,82]. From an economic perspective, it is important to determine whether the costly tailoring of games yields better results compared with conventional or commercial interventions. Further research is needed to address this question.

### Limitations and Implications for Future Research

The current meta-analysis has several limitations that should be considered when interpreting the findings. First, a notable limitation is the lack of adherence to open science principles, particularly the absence of preregistration before conducting the research.

Second, evidence for publication bias was found among the studies included in this analysis. This suggests that the findings may not fully represent the true effects due to a potential overrepresentation of studies with positive results [83,84]. However, when studies with a high risk of bias were excluded, the asymmetry was no longer significant, indicating that publication bias was not evident in the remaining studies.

Third, the included studies exhibited substantial heterogeneity concerning the target group, interventions, software used, and intervention duration. Although we utilized a random-effects model to account for this variability, considerable heterogeneity remained in the findings. Our sensitivity and moderator analyses managed to reduce, but not entirely resolve, this heterogeneity. Potential sources of heterogeneity that were not examined are the type of software used for the interventions, whether fatigue was a primary or secondary outcome, and intervention intensity and frequency, rather than just duration. We opted to focus on duration because this information was available for all studies.

Fourth, the overall number of studies was rather limited, covering a small variety of chronic diseases and interventions. This limitation was particularly pronounced for studies involving children, which is concerning given that up to 21% of children with chronic disease experience severe fatigue [85]. The need for effective treatment in this population is as urgent as it is for adults. Additionally, no high-quality studies were available for analysis, as determined by the Cochrane risk of bias assessment tool. However, it is worth noting that this tool has been reported to have relatively low reliability [86] and is considered more conservative compared with other risk of bias assessments [87].

Fifth, adherence to the study protocol and treatment satisfaction were not systematically measured nor compared with conventional active intervention groups. This aspect is crucial for determining whether game-based interventions are indeed more motivating than their conventional counterparts and should be a focus of future studies.

Sixth, on a more technical note, different measurement scales for fatigue were used across studies. One study used the VAS to measure fatigue, which is methodologically suboptimal as it is not specifically developed or validated for fatigue assessment and does not differentiate between various aspects of fatigue. To minimize the impact of this on the results, a second analysis was conducted, excluding the study that used VAS. The result remained significant, although the effect size was smaller. This suggests that while the VAS had a substantial influence on the outcome, it was not the sole contributor, as the significant effect appears robust. Although we attempted to mitigate potential discrepancies by standardizing outcome measures using the SMD, variations in psychometric properties may have influenced the results within the studies themselves. Additionally, we had to impute SDs for 1 study [58], means and SDs for 3 studies [53,57,60], and pre-post correlation for 14 studies [45,46,52-55,58-65]. This introduces a degree of uncertainty to our findings, as the reliability of these estimates is uncertain.

Finally, the findings presented here reflect short-term outcomes. As most of the studies did not include follow-up measures, we are unable to draw any conclusions about the long-term efficacy of game-based eHealth interventions.

Overall, more studies are needed across all age groups and various chronic diseases where fatigue is a side effect, to better determine whether these interventions are suited for each disease. These studies should adhere to rigorous design and methodology, including follow-up measures, to assess long-term treatment effects and the use of an intention-to-treat analysis approach for data analysis. We recommend testing not only commercial games but also developing more tailored and personalized games that allow for the investigation of treatments beyond physical activity. In particular, a combination of psychological interventions and physical activity is warranted [18].

### Conclusions

Based on the current meta-analysis, we cannot yet make clear recommendations for the use of eHealth interventions in clinical practice. However, we can cautiously conclude that eHealth interventions are effective in reducing fatigue in chronic diseases. As the number of studies in this field is steadily increasing, we hope to soon be able to back up our findings and extend them to other chronic conditions as well.

## Appendix

Multimedia Appendix 1 Literature search protocol.

Multimedia Appendix 2 PRISMA checklist.

## Abbreviations

EDSS: Expanded Disability Status Scale
MS: multiple sclerosis
POMS-sf: Profile of Mood States—short form
SMD: standardized mean difference
VAS: Visual Analog Scale
VR: virtual reality
