# Supplementary material for: Game-Based eHealth Interventions for the Reduction of Fatigue in People With Chronic Diseases: Systematic Review and Meta-Analysis
Source: JMIR Serious Games. 2024 Oct 17;12:e55034. doi: 10.2196/55034 (PMC11528177; doi:10.2196/55034)
Supplement: Multimedia Appendix 1 [file games_v12i1e55034_app1.docx]

Literature search protocol

**1^st^ round: 25^th^ August 2021**

| **Database searched** | **via** | **Years of coverage** | **Records** | **Records after duplicates removed** |
| --- | --- | --- | --- | --- |
| Embase | Embase.com | 1971 - Present | 866 | 850 |
| Medline ALL | Ovid | 1946 - Present | 432 | 77 |
| PsycINFO | Ovid | 1806 - Present | 196 | 92 |
| Web of Science Core Collection* | Web of Knowledge | 1975 - Present | 916 | 510 |
| Cochrane Central Register of Controlled Trials | Wiley | 1992 - Present | 313 | 119 |
| Other sources: Google Scholar | | | 200 | 94 |
| **Total** | | | **2923** | **1742** |

*Science Citation Index Expanded (1975-present) ; Social Sciences Citation Index (1975-present) ; Arts & Humanities Citation Index (1975-present) ; Conference Proceedings Citation Index- Science (1990-present) ; Conference Proceedings Citation Index- Social Science & Humanities (1990-present) ; Emerging Sources Citation Index (2015-present)

**Embase.com**

('game'/de OR 'recreational game'/exp OR 'serious game'/de OR 'virtual reality'/exp OR (((serious* OR exer* OR active* OR educat* OR computer* OR digital* OR mobile* OR video* OR online* OR phone* OR smartphone*) NEAR/3 (game* OR gaming)) OR exergam* OR Nintendo* OR Wii OR Kinect OR Xbox OR playstation OR VR OR virtual-realit* OR virtual-environment* OR videogam*):ab,ti,kw) **AND** ('fatigue'/exp OR (fatigue* OR tired* OR lassitude* OR lack-of-energ* OR exhaustion*):ab,ti,kw)

**Medline (ovid)**

(Games, Recreational/ OR Video Games/ OR Virtual Reality/ OR (((serious* OR exer* OR active* OR educat* OR computer* OR digital* OR mobile* OR video* OR online* OR phone* OR smartphone*) ADJ3 (game* OR gaming)) OR exergam* OR Nintendo* OR Wii OR Kinect OR Xbox OR playstation OR VR OR virtual-realit* OR virtual-environment* OR videogam*).ab,ti,kf.) **AND** (exp Fatigue/ OR (fatigue* OR tired* OR lassitude* OR lack-of-energ* OR exhaustion*).ab,ti,kf.)

**PsycINFO**

(Games/ OR Computer Games/ OR Virtual Reality/ OR (((serious* OR exer* OR active* OR educat* OR computer* OR digital* OR mobile* OR video* OR online* OR phone* OR smartphone*) ADJ3 (game* OR gaming)) OR exergam* OR Nintendo* OR Wii OR Kinect OR Xbox OR playstation OR VR OR virtual-realit* OR virtual-environment* OR videogam*).ab,ti.) **AND** (Fatigue/ OR (fatigue* OR tired* OR lassitude* OR lack-of-energ* OR exhaustion*).ab,ti.)

**Cochrane Central**

((((serious* OR exer* OR active* OR educat* OR computer* OR digital* OR mobile* OR video* OR online* OR phone* OR smartphone*) NEAR/3 (game* OR gaming)) OR exergam* OR Nintendo* OR Wii OR Kinect OR Xbox OR playstation OR VR OR virtual NEXT realit* OR virtual NEXT environment* OR videogam*):ab,ti,kw) **AND** ((fatigue* OR tired* OR lassitude* OR lack NEXT of NEXT energ* OR exhaustion*):ab,ti,kw)

**Web of Science**

TS=(((((serious* OR exer* OR active* OR educat* OR computer* OR digital* OR mobile* OR video* OR online* OR phone* OR smartphone*) NEAR/2 (game* OR gaming)) OR exergam* OR Nintendo* OR Wii OR Kinect OR Xbox OR playstation OR VR OR virtual-realit* OR virtual-environment* OR videogam*)) **AND** ((fatigue* OR tired* OR lassitude* OR lack-of-energ* OR exhaustion*)))

**Google Scholar**

"serious|active|education|educational|computer|digital|mobile|video|online|phone|smartphone game|games|gaming"|exergaming|Nintendo|Wii|Kinect|Xbox|playstation|"virtual reality|environment" fatigue|tired|tiredness

**2^nd^ round: 2^nd^ March 2023**

| **Database searched** | **via** | **Years of coverage** | **Records** | **Records after duplicates removed** |
| --- | --- | --- | --- | --- |
| Embase | Embase.com | 1971 - Present | 1113 | 1099 |
| Medline ALL | Ovid | 1946 - Present | 552 | 106 |
| PsycINFO | Ovid | 1806 - Present | 241 | 114 |
| Web of Science Core Collection* | Web of Knowledge | 1975 - Present | 1211 | 677 |
| Cochrane Central Register of Controlled Trials | Wiley | 1992 - Present | 424 | 198 |
| Other sources: Google Scholar | | | 200 | 74 |
| **Total** | | | **3741** | **2268** |

*Science Citation Index Expanded (1975-present) ; Social Sciences Citation Index (1975-present) ; Arts & Humanities Citation Index (1975-present) ; Conference Proceedings Citation Index- Science (1990-present) ; Conference Proceedings Citation Index- Social Science & Humanities (1990-present) ; Emerging Sources Citation Index (2015-present)

**Embase.com**

('game'/de OR 'recreational game'/exp OR 'serious game'/de OR 'virtual reality'/exp OR (((serious* OR exer* OR active* OR educat* OR computer* OR digital* OR mobile* OR video* OR online* OR phone* OR smartphone*) NEAR/3 (game* OR gaming)) OR exergam* OR Nintendo* OR Wii OR Kinect OR Xbox OR playstation OR VR OR virtual-realit* OR virtual-environment* OR videogam*):ab,ti,kw) **AND** ('fatigue'/exp OR (fatigue* OR tired* OR lassitude* OR lack-of-energ* OR exhaustion*):ab,ti,kw)

**Medline (ovid)**

(Games, Recreational/ OR Video Games/ OR Virtual Reality/ OR (((serious* OR exer* OR active* OR educat* OR computer* OR digital* OR mobile* OR video* OR online* OR phone* OR smartphone*) ADJ3 (game* OR gaming)) OR exergam* OR Nintendo* OR Wii OR Kinect OR Xbox OR playstation OR VR OR virtual-realit* OR virtual-environment* OR videogam*).ab,ti,kf.) **AND** (exp Fatigue/ OR (fatigue* OR tired* OR lassitude* OR lack-of-energ* OR exhaustion*).ab,ti,kf.)

**PsycINFO**

(Games/ OR Computer Games/ OR Virtual Reality/ OR (((serious* OR exer* OR active* OR educat* OR computer* OR digital* OR mobile* OR video* OR online* OR phone* OR smartphone*) ADJ3 (game* OR gaming)) OR exergam* OR Nintendo* OR Wii OR Kinect OR Xbox OR playstation OR VR OR virtual-realit* OR virtual-environment* OR videogam*).ab,ti.) **AND** (Fatigue/ OR (fatigue* OR tired* OR lassitude* OR lack-of-energ* OR exhaustion*).ab,ti.)

**Cochrane Central**

((((serious* OR exer* OR active* OR educat* OR computer* OR digital* OR mobile* OR video* OR online* OR phone* OR smartphone*) NEAR/3 (game* OR gaming)) OR exergam* OR Nintendo* OR Wii OR Kinect OR Xbox OR playstation OR VR OR virtual NEXT realit* OR virtual NEXT environment* OR videogam*):ab,ti,kw) **AND** ((fatigue* OR tired* OR lassitude* OR lack NEXT of NEXT energ* OR exhaustion*):ab,ti,kw)

**Web of Science**

TS=(((((serious* OR exer* OR active* OR educat* OR computer* OR digital* OR mobile* OR video* OR online* OR phone* OR smartphone*) NEAR/2 (game* OR gaming)) OR exergam* OR Nintendo* OR Wii OR Kinect OR Xbox OR playstation OR VR OR virtual-realit* OR virtual-environment* OR videogam*)) **AND** ((fatigue* OR tired* OR lassitude* OR lack-of-energ* OR exhaustion*)))

**Google Scholar**

"serious|active|education|educational|computer|digital|mobile|video|online|phone|smartphone game|games|gaming"|exergaming|Nintendo|Wii|Kinect|Xbox|playstation|"virtual reality|environment" fatigue|tired|tiredness
